# Liposomes as delivery vectors for nucleic acid mimics into *Escherichia coli*: effect of composition and functionalization

**DOI:** 10.3389/fbioe.2026.1850483

**Published:** 2026-05-28

**Authors:** João P. Santos, Eva Pinho, Rita S. Santos, Joana A. Loureiro, Maria C. Pereira, Rui M. Ferreira, Céu Figueiredo, Emílio Gomes, Joana Castro, Daniela Araújo, Nuno M. Guimarães, Nuno F. Azevedo

**Affiliations:** 1 LEPABE – Laboratory for Process Engineering, Environment, Biotechnology and Energy, ALiCE – Associate Laboratory in Chemical Engineering, Faculty of Engineering, University of Porto, Porto, Portugal; 2 INIAV - National Institute for Agrarian and Veterinary Research, Vairão, Portugal; 3 Department of Mechanical Engineering, Faculty of Engineering, University of Porto, Porto, Portugal; 4 i3S – Instituto de Investigação e Inovação em Saúde, Universidade do Porto, Porto, Portugal; 5 Faculty of Medicine, University of Porto, Porto, Portugal

**Keywords:** antimicrobial resistance (AMR), ASOs, *Escherichia coli*, liposomes, NAMs, polyclonal antibodies

## Abstract

The growing threat of antimicrobial resistance (AMR) requires the development of novel therapeutic solutions and Nucleic Acid Mimics (NAMs) arepresent a promising alternative. NAMs can hybridize with intracellular bacterial mRNA, working as antisense oligonucleotides (ASOs), being therefore potentially applied as targeted therapeutics (ASObiotics). Because NAMs do not naturally penetrate the bacterial envelope, effective delivery vectors are required. Liposomes represent a promising strategy to overcome this barrier, but their interaction with bacterial envelopes remains poorly understood. Here, we evaluated multiple liposomal formulations as delivery vectors for Cy3-labelled LNA/2′-O-methyl NAMs, targeting the essential *acpP* bacterial gene. Liposomes were engineered using different pairings of cationic/ionizable and helper lipids, a PEG-conjugated stabilizing lipid, and anti-*Escherichia coli* (*E. coli*) polyclonal antibodies. A DOTAP/DOPE formulation at 75:25 M ratio achieved over 80% delivery efficiency in *E. coli*. However, the inclusion of DSPE-methoxy-PEG resulted in reduced efficiency. The conjugation of anti-*E. coli* polyclonal antibodies significantly enhanced species-specific delivery, reducing labeling efficiency in non-target bacteria by approximately 20%. Higher antibody amounts improved delivery efficiency and specificity. The formulations showed no detectable cytotoxicity *in vivo* using the *Galleria mellonella* infection model. These findings demonstrate that antibody-functionalized liposomes provide an effective and selective platform for NAM delivery to bacterial cells.

## Graphical Abstract

**Figure:**
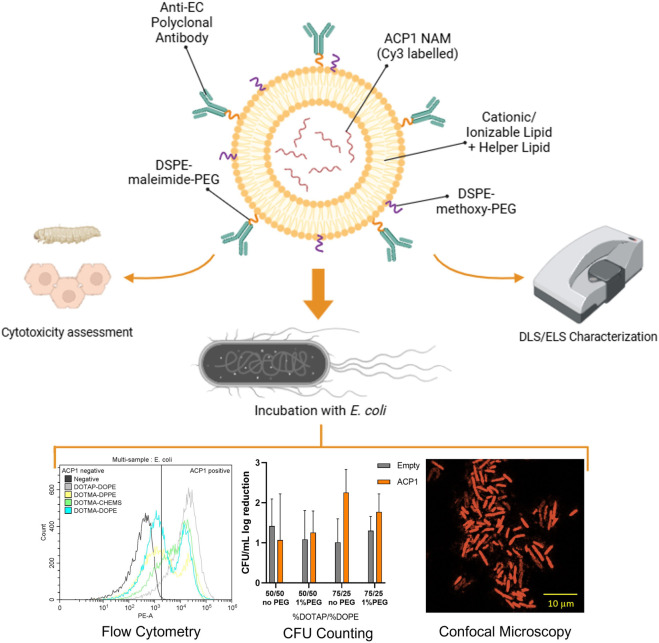

## Introduction

The development of resistance by bacteria coupled with the slow development of new antibiotics has been having devastating effects across the globe, with the estimated number of annual deaths surpassing one million (Naghavi et al., 2024). The “post-antibiotic era” is fully established and it is urgent to develop new therapeutic solutions to combat Multi Drug Resistant (MDR) bacteria, as reported (and continuously updated) by the World Health Organization (WHO) (WHO, 2026) and the Food and Drug Administration (FDA) (FDA, 2026).

One of the most promising solutions for both treatment and diagnosis of bacterial infections is the application of nucleic acid mimics (NAMs) (Santos et al., 2017). NAMs feature higher nuclease resistance, enhanced affinity/selectivity to their target mRNA sequences and improved thermodynamic properties (compared to natural occurring nucleic acids), due to alterations in the sugar rings and/or phosphodiester backbone (Santos et al., 2017; Karkare and Bhatnagar, 2006). Furthermore, these molecules are easily modified to keep up with the new genetic variants of resistant strains, circumventing that same resistance and maintaining their efficiency (Hegarty and Stewart, 2018). Bacterial genome sequencing can be used to identify resistance-related mutations, allowing the “correction” of an obsolete NAM molecule. NAMs can therefore work as enhanced antisense oligonucleotides (ASOs) and potentially be applied as alternative therapeutics.

The main obstacle to the development of ASO based antibiotics (ASObiotics) is the poor internalization of these molecules in bacterial cells due to their complex cell envelope, necessitating the use of vectors able to deliver the NAMs across the multi-layered bacterial envelope and into the bacterial cytosol (Santos et al., 2017; Guo et al., 2006; Santos et al., 2018).

Liposomes are among the most studied and biocompatible delivery vectors in eukaryotic cells, including for transport of antibiotics, to protect them from degradation and to enable their presence at higher concentrations at the site of action (Schiffelers et al., 2001; Omri and Ravaoarinoroa, 1996). The potential of liposomes to cross the cell envelope of bacteria and deliver antimicrobial compounds into the bacterial cytosol has started being addressed, although further studies are still needed to better understand this interaction and improve delivery efficiency (Santos et al., 2018; Drulis-kawa et al., 2006).

Phosphatidylethanolamine (PE) is the most reported fusogenic lipid, as it destabilizes membranes through promotion of the transition between bilayer and hexagonal phases (Haque et al., 2001; Simões et al., 2001). For this reason, it is commonly used in liposomes, in the form of dioleoylphosphatidylethanolamine (DOPE), to confer the desired fusogenic properties (Nicolosi et al., 2010; Ma et al., 2013). Dioleoyl-3-trimethylammonium propane (DOTAP) is often used in conjugation with other lipids, as its positive charge enhances the interaction with negatively charged cellular membranes (Drulis-kawa et al., 2009). Polyethylene glycol (PEG)-lipids are also commonly used in a small percentage, to improve the mobility and colloidal stability of the liposomes through the different harsh environments they face *in vivo* when used as therapeutics (Santos et al., 2017; Pereira et al., 2021a).

Studies involving eukaryotic cells point to the electrostatic interaction between the positive charges of cationic liposomes (composed of one cationic and one neutral helper lipid) and the negative charges of cell membrane sulfated proteoglycans as an important mechanism for the cell-liposome binding (Simões et al., 2005). Similarly, cationic liposomes interact with the negatively charged cell wall of both Gram-positive and Gram-negative bacteria (Santos et al., 2018; Drulis-kawa et al., 2009).

We have previously applied LipoNAMs (LN) composed of DOTAP, DOPE and DSPE-PEG for labelling of human microbiota with NAMs, achieving greater efficiency than the typical fixation/permeabilization methods used *in vitro* in Fluorescence *In Situ* Hybridization (FISH) (Moreira et al., 2022). The ability of post-PEGylated DOTAP/DOPE liposomes to fuse with both Gram-negative and Gram-positive bacteria and deliver LNA/2′OMe mixmers was also demonstrated (Santos et al., 2017).

More recently, ImmunoLiposomes (IL) were synthesized and reported to be capable of targeting specific receptors in a cell model of the blood-brain barrier (Loureiro et al., 2015) and to improve the selectivity and biocompatibility in the delivery of NAMs into bacteria (Moreira et al., 2023).

The assessment of liposome formulations is critical for their application as a vector for the delivery of nucleic acids into bacteria and the specificity of that delivery is also fundamental, as the natural microbiomes must not be affected. Also, a higher specificity of the delivery will increase the NAM concentration at the targeted bacteria, consequently improving the antibacterial effect. In this work, the effect of different lipid compositions/concentrations on the delivery efficiency of NAMs into *Escherichia coli* (*E. coli*) by fusogenic cationic liposomes was assessed. Furthermore, species-specific antibodies were conjugated to the LipoNAMs with the best performance to enhance the construct’s specificity. All developed formulations were evaluated in terms of delivery efficiency, selectivity and effect on bacterial cell viability. *In vitro* and *in vivo* cytoxicity assays were also performed.

## Materials and methods

### Bacterial cultures

*Escherichia coli* CSH36 (a K12 mutant, Coli Genetic Stock Center, Yale University, CT, USA), *E. coli* ATCC25922, *E. coli* CECT740, *E. coli* CECT832, *Salmonella enterica* Typhimurium SGSC2523, *S. enterica* Typhimurium SGSC1420, *S. enterica* Typhimurium SGSC3029, *Listeria monocytogenes* ATCC7644 and *L. monocytogenes* CECT5873 were harvested from Tryptic Soy Agar (TSA) (Agar from VWR International bv Geldenaaksebaan, Leuven, Belgium) plates and inoculated into 10 mL Tryptic Soy Broth (TSB) (Merck KGaA, 64271 Darmstadt, Germany). Then, bacteria were incubated for 16–18 h at 37 °C with shaking at 180 rpm. The cultures were then diluted in Tryptic Soy Broth to an optical density (OD) at 600 nm of 0.1. After 2 h of regrowth under the previously referred conditions to reach the logarithmic growth phase, the cultures were diluted once more to OD_600_ = 0.1 (corresponding to approximately 5 × 10^9 CFU/mL), prior to the experiments.

### Liposome synthesis

Liposomes were prepared by the ethanol dilution method described by Jeffs et al. (2005) with slight alterations and using the pre-PEGylation method. A cationic/ionizable lipid, a helper lipid and DSPE-methoxy-PEG (Avanti Polar Lipids, Alabaster, AL, USA) were mixed at the pre-determined molar ratios/percentages to prepare PEGylated liposomes. To prepare pre-PEGylated maleimide-functionalized liposomes (Mal-LN), different molar percentages of DSPE-maleimide-PEG (Avanti Polar Lipids, Alabaster, AL, USA) and 1 mol% DSPE-methoxy-PEG were mixed with the main lipids. Briefly, the lipids were dissolved in chloroform, and the solvent was evaporated under a nitrogen stream. The lipids were redissolved in 90% ethanol to a final lipid concentration of 40 mM. The ethanolic solution was mixed with an equal volume of 4 μM ACP1 oligonucleotide (Eurogentec, Belgium) in ultra-pure H_2_O to prepare LipoNAMs, agitated manually for 20 s and then incubated at room temperature for 5 min. Unloaded liposomes were prepared by mixing the ethanolic solution with HEPES buffer (10 mM HEPES hemisodium salt, pH = 7.4, Sigma-Aldrich, Lisbon, Portugal). Ethanol was removed by ultrafiltration using an Amicon® ultra-0.5 30K centrifugal filter device (Merck Millipore, Burlington, MA, USA). Pre-PEGylated liposomes were then diluted in HEPES to a lipid concentration of 5 mM.

Free ACP1 in liposome suspensions was removed by a PD SpinTrap™ G-25 column (GE Healthcare, Pittsburgh, PA, USA) and the fluorescence intensity was measured using a microtiter reader FLUOstar OMEGA (BMG Labtech, Ortenberg, Germany), equipped with filter ex485–12/em520–10, before and after unloaded ACP1 removal. To estimate the loading efficiency, Equation 1 was used:

$$\%\text{LE}=\left[\text{loaded ACP}1\right]/\left[\text{total ACP}1\right]*100$$ (1)

where %LE is the loading efficiency and [loaded ACP1] and [total ACP1] is the concentration of ACP1 after and before unloaded ACP1 removal, respectively.

The liposomes’ hydrodynamic diameter, polydispersity index (PDI) and zeta-potential were routinely controlled by dynamic light scattering (DLS, Malvern ZetaSizer instrument, Malvern Instruments Ltd., Malvern, UK).

### Preparation of ImmunoLiposomes/ImmunoLipoNAMs

Antibody-conjugated liposomes were prepared following the protocol described by Loureiro et al. (Loureiro et al., 2015) with some modifications. Briefly, 0.02 mM rabbit anti-*E. coli* polyclonal antibody (Bio-Rad 4329–4906, BIO-RAD Laboratories, Amadora, Portugal) was incubated with 0.4 mM 2-iminothiolane (in 0.044 mM PBS) in the presence of 5 mM ethylenediaminetetraacetic acid (EDTA) for 1 h at room temperature. Then, antibodies were purified through a PD SpinTrap™ G-25 column (GE Healthcare, Pittsburgh, PA, USA) equilibrated with sterile milli-Q water. Maleimide-functionalized liposomes at 20 mM (DOTAP/DOPE concentration) were immediately mixed with purified antibodies to 1:10 or 1:5 antibody-to-maleimide molar ratio and incubated for 1 h at room temperature, followed by a second incubation at 4 °C overnight. The hydrodynamic size was routinely controlled by dynamic light scattering (Malvern ZetaSizer instrument, Malvern Instruments Ltd., Malvern, UK). Antibody-conjugated liposomes were diluted in HBSS before the experiments with bacteria.

### Delivery efficiency assay

The delivery efficiency of LipoNAMs was evaluated according to the experimental procedure described by Santos et al. (2017) with some modifications. Briefly, bacteria in the exponential growth phase (1 mL) were centrifuged at 16,800 × *g* for 5 min and resuspended in 50 μL of HEPES buffer or 5 mM LipoNAMs, followed by incubation at 37 °C for 1 h. Bacteria were then pelleted by centrifugation at 10,000 x *g* for 5 min and rinsed with 500 μL of pre-warmed (37 °C) stringent solution (15 mM NaCl, 0.1% Triton-X (v/v), 5 mM Tris Base, pH = 10) for 15 min at 37 °C to wash liposomes adsorbed on the cells’ surface. Finally, bacteria were pelleted by centrifugation at 10,000 × *g* for 5 min and resuspended in 1 mL of sterile milli-Q water before sonication in a bath sonicator at room temperature for 10 min. The internalization of ACP1 in bacteria was quantified by flow cytometry using a CytoFLEX model V0-B3-R1 (Beckman Coulter, Brea, CA, USA) with CytExpert software (version 2.4.0.28) and observed under a Nikon Eclipse T*i* SR Inverted Microscope.

**FIGURE 1:**
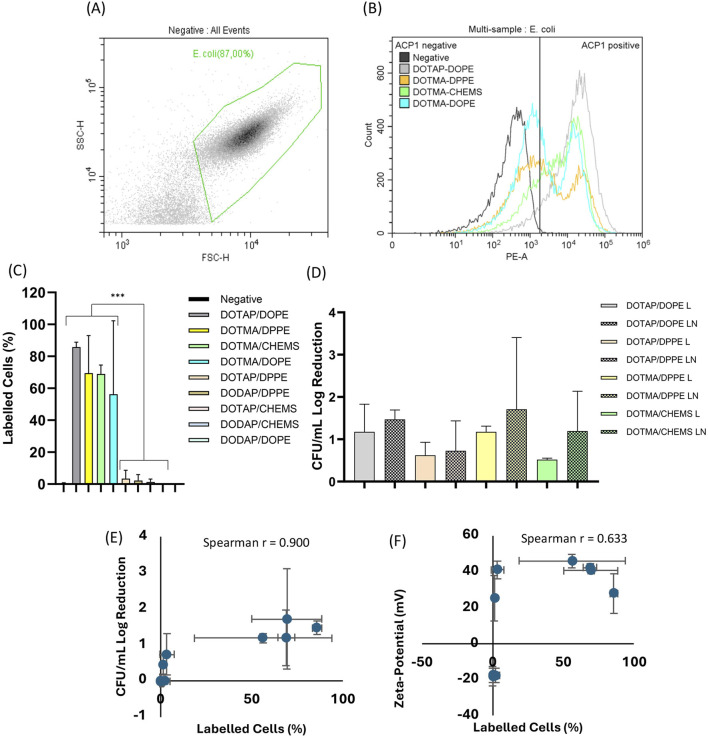
**(A)** Gating of the *Escherichia coli* CSH36 population. **(B)** Graphic example of the cytometry data, with the user-defined threshold for negative/positive ACP1 labelling. **(C)** Percentage of *Escherichia coli* cells exhibiting a positive ACP1 staining determined by flow cytometry after 1 h in contact with LipoNAMs with 75:25 M ratio of cationic/helper lipid and 1% DSPE-methoxy-PEG. **(D)** Culturability (expressed as CFU/mL log reduction) of *Escherichia coli* ATCC25922 after 1 h of exposure to different liposome and LipoNAM formulations. **(E)** Correlation plot between culturability (CFU/mL log reduction) and labelling efficiency (*Escherichia coli* labelled cells %) after 1 h of incubation with different LipoNAM formulations. **(F)** Correlation plot between superficial charge of LipoNAMs (Zeta-Potential) and labelling efficiency (*Escherichia coli* labelled cells %) after 1 h of incubation with different LipoNAM formulations.

The forward and side scatter of a negative sample (bacteria treated only with HEPES buffer) are used to select the target population. An example of this selection is shown in Figure 1A. This gating allows cell labelling to be assessed more precisely, while excluding debris that may be present in the sample (which are kept outside the defined gate). The data was collected for 30,000 events inside this gate.

The NAMs encapsulated in the different liposomes are labelled with orange Sulfo-Cyanine3 (Cy3). Hence, the PE channel of the cytometer is selected for detection, using the 488 nm argon excitation laser (50 mW) and a 585/42 band-pass filter.

### Bacterial culturability assay (CFU counting)

The bacterial culturability of different strains after contact with various liposomal formulations was determined through Colony Forming Units (CFU) counting. Briefly, bacteria in the exponential growth phase (1 mL) were centrifuged at 16,800 × *g* for 5 min and resuspended in 50 μL of HEPES buffer (negative) or 5 mM LipoNAMs followed by incubation at 37 °C for 1 h. A 10 μL sample was collected and diluted in saline solution (0.85%). Serial dilutions of each condition were prepared, and three drops (10 μL) were plated in Tryptic Soy Agar (TSA) plates. After *overnight* incubation at 37 °C, the CFUs were counted, and the logarithmic reduction was determined by comparing the conditions to the negative.

### MTS assay

The cytotoxicity of constructs was tested using the HepG2 cell line (hepatoblastoma, DSMZ-German Collection of Microorganisms and Cell Cultures, GmbH). Cells were cultured in Roswell Park Memorial Institute Medium (RPMI)-1640 medium with stable glutamine and supplemented with penicillin-streptomycin (100 IU/mL penicillin, 100 mg/mL streptomycin, Biowest, USA) and heat-inactivated fetal bovine serum (Biowest, USA) 10% (v/v). The viability of cells was determined using CellTiter 96® AQueous One Solution Cell Proliferation Assay (Promega, Madison, WI, USA), following the manufacturer’s recommendations. HepG2 cells were grown in T25 culture flasks and detached (Thermo Fisher Scientific, USA) using trypsin, transferred to 96-well microplates at 5 × 10^4^ cells/well, and incubated at 37 °C in a 5% CO_2_ atmosphere for 48 h. The medium was removed from the wells and 100 µL of the different liposome formulations (5 mM of lipids, 500 nM of ACP1) in FBS-free medium were added and incubated for 24 h under the same conditions. Buffer in FBS-free medium and 50% (v/v) DMSO (Sigma-Aldrich, Lisbon, Portugal) in PBS were used as negative and positive controls, respectively. The suspensions were then removed and CellTiter 96® AQueous One Solution Cell Proliferation Assay (Promega, Madison, WI, USA) diluted in fresh complete medium was added to sterile (blank) or cell-containing wells. The microplates were incubated for 4 h at 37 °C in a 5% CO_2_ atmosphere before absorbance reading at 490 nm and 650 nm in a Bio-Rad Model 680 microplate reader (Bio-Rad Laboratories, Lda, Portugal). Both A_test well_ and A_control well_ are defined as (A_490nm_–A_650nm_), as 650 nm is the reference wavelength to subtract background contributed by nonspecific absorbance. The viability was calculated using Equation 2:

$$\text{Viability }\left(\%\right)=\left(A_{\text{test}\;\text{well}}\right)/\left(A_{\text{control}\;\text{well}}\right)*100$$ (2)

where, Viability (%) is the percentage of cells viability, A_test well_, and A_control well_ are the absorbances of test cells and buffer-exposed cells, respectively.

### Galleria mellonella viability assay

*Galleria mellonella* larvae were maintained at 25 °C, in the dark, and following a diet of pollen grains. Larvae were used at the final larval stage, when they weighed approximately 250 mg. In each assay, a set of ten selected larvae was injected with 10 μL of the sample under study (5 mM lipids, 500 nM ACP1) in the hindmost left proleg, previously sanitized with 70% (v/v) ethanol. Following injection, larvae were placed in Petri dishes and incubated in the dark at 37 °C. Larval survival was monitored for a period of 3 days (24, 48, and 72 h post-infection). Larvae that did not respond to touch were considered dead. The *G. mellonella* health index, which assesses four main parameters, was also determined: larval activity, cocoon formation, melanisation, and survival, according to a pre-established score system (Supplementary Table S3) (Loh et al., 2013). To ensure reliable results, all experiments were performed at least in triplicate, independently. Ethical approval is not required for the study of this animal model.

### Statistical analysis

Statistical analysis was performed using GraphPad Prism8 software (GraphPad Software, San Diego, CA, USA). All results were analyzed by one-way or two-way ANOVA and *post hoc* Tukey’s multiple comparisons test. Data were plotted as mean ± standard deviation of three independent assays. Statistically differences were set as *P < 0.05; **P < 0.01; ***P < 0.001; ****P < 0.0001. Correlations between variables were evaluated using the Spearman’s rank correlation coefficient.

## Results and discussion

### Testing different combinations of cationic/Ionizable and helper lipids

**FIGURE 2:**
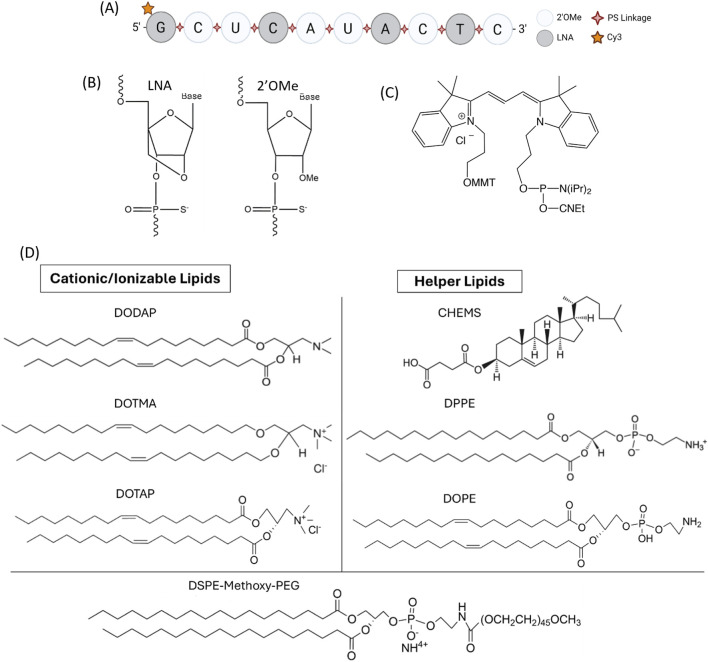
**(A)** Structure and sequence of ACP1 oligonucleotide, with identification of the LNA and 2′-OMe nucleotides and presence of PS linkages. **(B)** Backbone modifications in ACP1 oligonucleotide. **(C)** Structure of the fluorophore sulfo-cianyne3 (Cy3). **(D)** Structure of the synthetic lipids used in liposomes formulations: 1,2-dioleoyl-3-dimethylammonium-propane (DODAP), cholesteryl hemisuccinate (CHEMS), 1,2-di-O-octadecenyl-3-trimethylammonium propane (DOTMA), 1,2-dipalmitoyl-sn-glycero-3-phosphoethanolamine (DPPE), 1,2-dioleoyl-3-trimethylammonium-propane (DOTAP), 1,2-dioleoyl-sn-glycero-3-phosphoethanolamine (DOPE) and 1,2-distearoyl-sn-glycero-3-phosphoethanolamine-N-[methoxy(polyethylene glycol)-2000] (DSPE-methoxy-PEG). **(A)** was created with biorender.com.

The ASO used in this work was the ACP1 (Figure 2A), which targets the mRNA of the essential fatty acid biosynthesis *acpP* gene (Nejad et al., 2021). This sequence was previously designed by our group (Pereira et al., 2021b) and consists of Locked Nucleic Acid (LNA) and 2′-O-Methyl RNA (2′OMe) nucleotides linked by phosphorothioate (PS) bonds (Figure 2B). The ACP1 oligonucleotide was labeled with Cy3 in the 5′ terminal to allow its detection by flow cytometry and microscopy. The molecular weight of Cy3 phosphoramidite (structure in Figure 2C) is 953.64 g/mol, accounting for 24.5% of the total weight of the ACP1 ASO.

Because of the need for cationic lipids for electrostatic interactions with the bacterial cells (Scheeder et al., 2023) and helper lipids for formulation stability and membrane fusion (Witzigmann et al., 2020), different pairs were tested using one of each type. Based on the results obtained previously, the combination of DOTAP and DOPE as major lipid components was included (Moreira et al., 2023). All combinations were applied in a 75:25 cationic/ionizable to helper lipid molar ratio. For stability purposes, DSPE-methoxy-PEG was included in all the formulations at 1 mol%. The structures of the selected lipids are presented in Figure 2D.

For all formulations, the loading efficiency of the ACP1 ASO was determined by measuring the fluorescence of LipoNAMs before and after the removal of the free ASO through filtration.

All combinations of lipids showed a loading efficiency above 80% (Supplementary Table S1), in accordance with the results obtained in a previous work (Moreira et al., 2023). The new formulations were also characterized through Dynamic Light Scattering (DLS) and Electrophoretic Light Scattering (ELS) (Supplementary Figure S3). The hydrodynamic size of the liposomes ranged from 158 to 508 nm, with average with Polydispersity Index (PDI) values between 0.251 and 0.587. The zeta-potential was positive for most of the formulations, as expected since cationic lipids were included, except for the ones including DODAP. This can be explained by the ionizable nature of the latter lipid, which does not assume a positive charge at neutral pH (Yan et al., 2024). Formulations whose properties were closer to the desired ones were prepared using the combinations DOTAP:DOPE and DOTAP:CHEMS.

The above-mentioned LipoNAMs were then applied to *E. coli*, and their internalization was tracked by flow cytometry and confocal microscopy. For flow cytometry, we started by using a pure culture of *E. coli* to identify the population of interest, when analyzing the forward and side scatters (Figure 1A). Bacterial cells were easily identified in the scatter plot, as they were the more complex and larger structures present in the sample. This sample was also used as a negative control for labelling.

Subsequently, after exposure of the bacterial cells to different liposomal formulations with labeled NAMs, we determined the percentage of labelled cells after defining the threshold for positive labelling (Figure 1B), which was determined using the negative sample mentioned above.

The delivery efficiency evaluation by flow cytometry (Figure 1C) of the 75:25 M ratio formulations revealed that the one using DOTAP and DOPE resulted in higher percentage of labelled cells, with less variability. For formulations containing the ionizable lipid DODAP, no significant cell labeling was observed. The formulations including the cationic DOTMA showed more variable labeling efficiencies, which can be attributed to increased variability in lipid vesicle properties (as seen in the PDI values, Supplementary Figure S1).

We then assessed bacterial culturability after 1 h of incubation with liposomes and LipoNAMs. While there are no statistically significant differences (p < 0.05) between the effect of empty liposomes and those loaded with ASOs, in some formulations the presence of the ASO resulted in a slightly greater decrease in culturability (Figure 1D). The logarithmic reductions achieved by the complete array of liposome formulations, as well as the results for a 4 h treatment, are presented in the Supplementary Material (Supplementary Figure S2) For 4 h, the reductions showed the same tendency across formulations and were only slightly higher, which can be explained by the rapid mechanism of action of the NAMs and the bacteria’s subsequent response as soon as the internalization is achieved. This behavior had already been observed by Popella et al. (2022).

A clear correlation (r = 0.900) was observed between the fluorescent labelling of *E. coli* and the decrease in their culturability (Figure 1E), which indicates a cause-effect relationship between the delivery of the NAM by liposomes and the antibacterial effect obtained. This also suggests that an antisense effect is occurring, indicating that bacterial death is not just caused by the toxicity of the lipid components. Another possible correlation that was observed was the one between the zeta-potential of LipoNAMs and the labelling of cells (r = 0.633) (Figure 1F). This highlights once more the important role of cationic lipids for interaction between vector and bacterial cells. We can, nevertheless, also conclude that superficial charge is not the only decisive factor for NAM delivery, since some formulations achieved low labelling efficiency while exhibiting high Zeta-Potential (e.g., DOTAP/DPPE).

These preliminary results led us to move forward with the DOTAP/DOPE combination. In addition to the higher labelling efficiency with lower standard deviations, the liposomes synthesized with this combination showed high zeta-potential and smaller sizes, crucial factors for the interaction with bacterial cells. It is important to note that sizes below 200 nm have been reported as necessary to prevent reticulo-endothelial rejection and allow penetration into infectious biofilms (Wang et al., 2020).

### Effect of PEGylation and main lipid ratio on the delivery efficiency

The presence of PEG in the liposomal formulation is fundamental for its stabilization and circulation lifetime (Li et al., 2014; Liang et al., 2005). However, it is important to consider the effect that this polymer may have on the capacity of the vesicles to deliver ASOs into bacterial cells. In fact, PEGylation is known to decrease delivery/transfection efficiency, as the aqueous layer it forms around the vesicle hampers the interaction with the cell surface (Hatakeyama et al., 2013; Gomes-Da-Silva et al., 2012).

We tested LipoNAMs with 50% of both DOTAP and DOPE and different DSPE-methoxy-PEG2000 M percentages (up to 1%) and assessed the delivery efficiency in *E. coli* by flow cytometry (Supplementary Figure S3). As expected, higher percentages of PEG lipid result in a clear decrease in *E. coli* labelling. These results are also in accordance with the ones published by Moreira et al. (2023), which show that both pre- and post-PEGylation of cationic fusogenic liposomes hamper the delivery of NAMs into *E. coli*.

The hydrodynamic size of the LipoNAMs, determined through DLS (results presented in Supplementary Figure S4), showed no significant variation with the different PEG percentages, ranging between 148.06 ± 16.81 nm and 137.93 ± 7.82 nm. The lower values of zeta potential with the increase of PEG percentage are explained by the neutralization of some cationic charges of DOTAP (Majzoub et al., 2014). The liposomes showed a PDI ranging from 0.322 to 0.384. The loading efficiency of ACP1 at different concentrations in liposomes with 50:50 M ratio of DOTAP:DOPE and 1 mol% DSPE-methoxy-PEG was determined by measuring the fluorescence before and after removal of unbound NAM and is presented in Supplementary Table S2).

**FIGURE 3:**
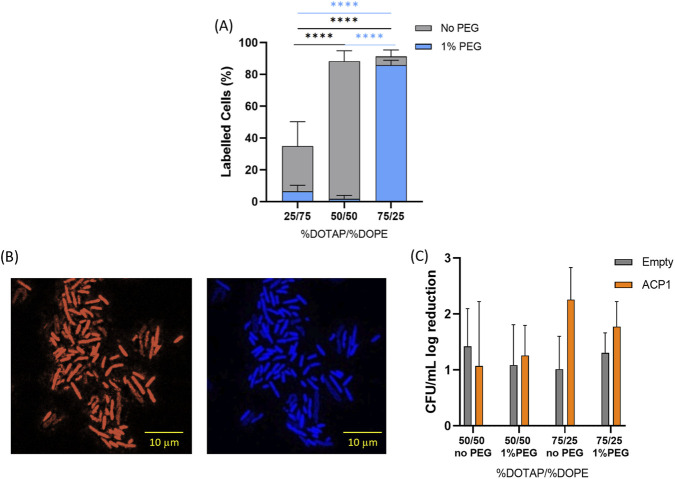
**(A)** Study of the influence of LipoNAMs main lipids composition in the delivery efficiency of NAMs to *Escherichia coli* CSH36. Percentage of cells exhibiting a positive ACP1 staining determined by flow cytometry after 1 h. **(B)** Confocal imaging of *Escherichia coli* CSH36, labelled using either ACP1 LipoNAMs (orange) or DAPI (blue). **(C)** Culturability (expressed as CFU/mL log reduction) of *Escherichia coli* CSH36 after 1 h exposure to different liposome formulations: 50:50 M ratio of DOTAP:DOPE with or without DSPE-methoxy-PEG (1%) and 75:25 of DOTAP:DOPE with or without DSPE-methoxy-PEG (1%). ****P < 0.0001.

Considering the low delivery efficiency of the tested PEGylated liposomes, different DOTAP:DOPE ratios were tested (75:25 and 25:75), adding to the 50:50 ratio, initially selected and previously applied by Moreira et al. (Moreira et al., 2023) The effect of these modifications in liposome composition on the delivery of NAMs in *E. coli* CSH36 is presented in Figure 3A. It is possible to conclude that a higher percentage of the cationic lipid DOTAP increases delivery efficiency, with PEGylated LipoNAMs achieving values similar to those obtained with the 50:50 ratio without PEG. This change in the ratio of main lipids allows the application of 1% DSPE-methoxy-PEG and the consequent stabilization of the vesicles without significantly affecting the ability to deliver their cargo into bacterial cells. The labelling of bacteria with the 75:25 ratio was also assessed qualitatively through Confocal Microscopy (Figure 3B), with general nucleic acid labelling (DAPI) for comparison.

To evaluate the antimicrobial activity of LipoNAMs, the culturability of *E. coli* CSH36 was evaluated after 1 h of contact with different formulations, including the two best main lipid ratios and the absence or presence (at 1 mol%) of DSPE-methoxy-PEG (Figure 3C). While the results are not statistically significant, there was still at least one logarithmic reduction in comparison with the negative control (HEPES buffer) for all formulations tested, while the LipoNAMs with a higher percentage of the cationic lipid DOTAP achieved a 2-log reduction without PEG and a 1.77-log reduction with 1 mol% PEG.

### Effect of the inclusion of DSPE-maleimide-PEG in the formulation

Since liposomes are a polyvalent, non-specific vector that can interact with virtually all bacteria, there is a need to introduce selectivity in the formulation. Therefore, the next step in this study consisted of conjugating polyclonal antibodies to improve the targeting of the delivery.

**FIGURE 4:**
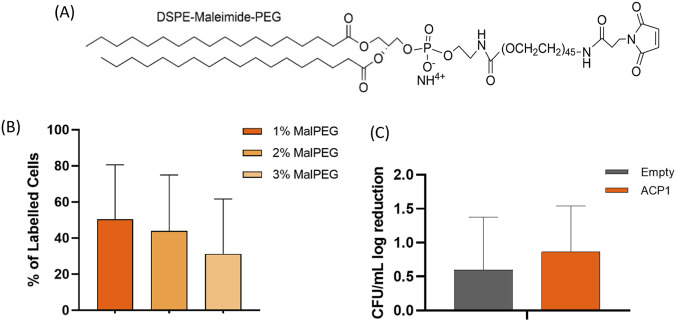
**(A)** Chemical structure of 1,2-distearoyl-sn-glycero-3-phosphoethanolamine-N-[maleimide(polyethylene glycol)-2000] (DSPE-maleimide-PEG). **(B)** Study of the influence of maleimide-PEG percentage in the delivery efficiency of NAMs to *Escherichia coli* CSH36 by liposomes with 75:25 DOTAP:DOPE ratio and 1% methoxy-PEG. Percentage of cells exhibiting a positive ACP1 staining determined by flow cytometry after 1 h. **(C)** Effect of LipoNAMs with 1% of each PEG lipid (1 h exposure) in the culturability of *Escherichia coli* CSH36.

For the synthesis of ImmunoLipoNAMs (ILN), two different PEG lipids were included in the formulation: methoxy-PEG for stabilization and maleimide-PEG (Figure 4A) for antibody conjugation (Loureiro et al., 2015). Thus, LipoNAMs with the best tested DOTAP:DOPE molar ratio (75:25), 1 mol% DSPE-methoxy-PEG2000 and different percentages of DSPE-maleimide-PEG2000 (Mal-LN) were tested (Figure 4B). From this assay, it was determined that the best ratio of lipids to use going forward was 75:25 of DOTAP:DOPE with 1 mol% of each of the PEG lipids. However, as opposed to what was verified by Moreira et al. (2023), LipoNAMs with only methoxy-PEG achieve better labelling of *E. coli* than Mal-LN. This can be a consequence of the different DOTAP:DOPE ratio and suggests that the superficial charge of the liposomes plays a more determinant role in the interaction with bacteria than the affinity of maleimide towards cell surface thiol groups.

The physical properties of LipoNAMs with different DSPE-maleimide-PEG percentages are presented in Supplementary Figure S5.

The bacterial viability assay was performed for the optimized formulation for antibody conjugation (Mal-LN): 75:25 M ratio of DOTAP:DOPE with 1 mol% of both DSPE-methoxy-PEG and DSPE-maleimide-PEG. The results show no statistically significant reduction in cell culturability compared to the control (Figure 4C). This decrease in the effect could result from the blockage of positive charges in DOTAP by the 2% of PEG lipids, although maleimide groups are reported to react with cell surface thiols (Moreira et al., 2023).

### Conjugation of antibodies to LipoNAMs improves the selectivity of the delivery

**FIGURE 5:**
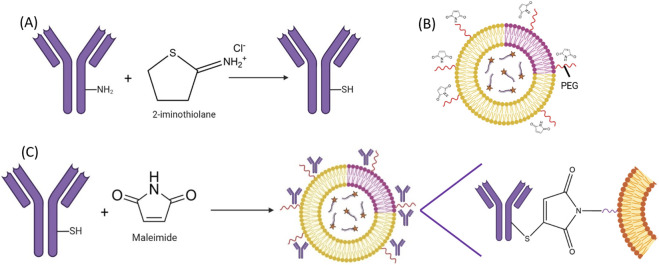
**(A)** Thiolation reaction of antibodies by reaction with Traut’s reagent (2-iminothiolane). **(B)** LipoNAM functionalized with DSPE-Maleimide-PEG. **(C)** Schematic representation of the conjugation of antibodies to LipoNAMs, through thiol-maleimide click reaction and formation of thioether bonds. Created with biorender.com, based on the illustration presented by Moreira et al. (2023).

To further explore the concept of PEGylated liposomes with affinity to specific species, ImmunoLipoNAMs were produced by the reaction of maleimide functional groups of Mal-LN with thiol-modified polyclonal antibodies targeting *E. coli* at different antibody-maleimide molar ratios. The liposome composition used was 75:25 of DOTAP:DOPE and 1 mol% of each of the PEG lipids and the synthesis process of these engineered liposomes is further detailed in Figure 5.

The ImmunoLipoNAMs were characterized through DLS (Supplementary Figure S6). The increase in hydrodynamic size and the decrease in zeta-potential in the formulation with higher antibody concentration indicate that the conjugation was successful. The presence of antibodies in the surface of lipid vesicles naturally increases their diameter and simultaneously blocks some of the positive charge conferred by DOTAP.

**FIGURE 6:**
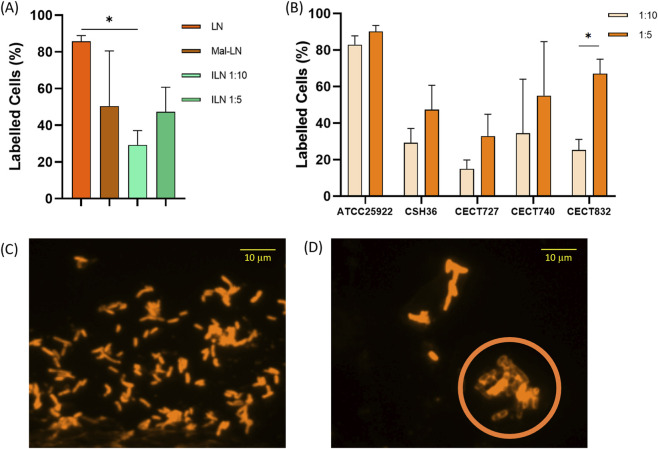
**(A)** Comparison between the different LipoNAMs formulations in terms of the delivery efficiency of NAMs into *Escherichia coli* CSH36. Percentage of cells exhibiting positive ACP1 staining determined by flow cytometry after 1 h. **(B)** Influence of antibody-maleimide ratios in the delivery efficiency of ImmunoLipoNAMs in different strains of *Escherichia coli*. Percentage of cells exhibiting positive ACP1 staining determined by flow cytometry after 1 h. **(C,D)** Epifluorescence images of *Escherichia coli* CSH36 after treatment with ImmunoLipoNAMs at 1:5 antibody-maleimide ratio and carrying 500 nM of ACP1 NAM. In **(D)**, the delineated area shows cells whose cytosol was not labelled by the fluorescently labeled nucleic acids. *P < 0.05.

The percentage of labelled cells was determined by flow cytometry and the results are presented in Figure 6A. The inclusion of antibodies (1:10 antibody-maleimide ratio) in the formulation reduced the delivery efficiency from 86% to 30%, but doubling the antibody concentration (1:5 antibody-to-maleimide ratio) improved the delivery back to 47%. This suggests that, although antibodies may block some electrostatic interactions between liposomes and the cell envelope, at higher concentrations they direct the construct to the *E. coli* specific antigens, resulting in a higher percentage of labelled cells. The labelling efficiency is lower than the one achieved by Moreira et al. (Moreira et al., 2023), but the exposure time is also significantly lower (1 h against 8 h), which may justify that difference. The influence of the antibody concentration is also contrasting, as that work reported a decrease in delivery with higher concentration. This may be explained by changes in the antigen target of the applied antibodies, namely, through phenomena such as antigenic variation, phase variation, and environmental adaptation (Lerouge and Vanderleyden, 2002).

ImmunoLipoNAMs in the two antibody-maleimide ratios were applied to different *E. coli* strains (Figure 6B). It can be concluded that, for the species targeted by the antibody, delivery is consistently favored by a higher antibody concentration.

Different strains of the same bacterial species may have different gene sets or different copy numbers of the same genes (Greenblum et al., 2015). Variability in the number of mRNA target copies may explain the differences observed in the delivery efficiency.

The delivery of NAMs was also evaluated through epifluorescence microscopy, with *E. coli* cells being consistently labelled by ImmunoLipoNAMs (Figure 6C). However, this labelling was sometimes seen only in the cell wall of bacteria and not in their cytosol (Figure 6D), which suggests that the NAM sequence might be retained in the complex barrier that is the bacterial cell wall. The variability in the fluorescence intensity of different cells may be due to the different number of copies of the target mRNA sequence (Skinner et al., 2013). mRNA targets are also present in lower concentration and are less stable than rRNA ones, typically used in FISH, which can justify the occasional low intensity fluorescence (Almeida and Azevedo, 2021; Coleman et al., 2007).

**FIGURE 7:**
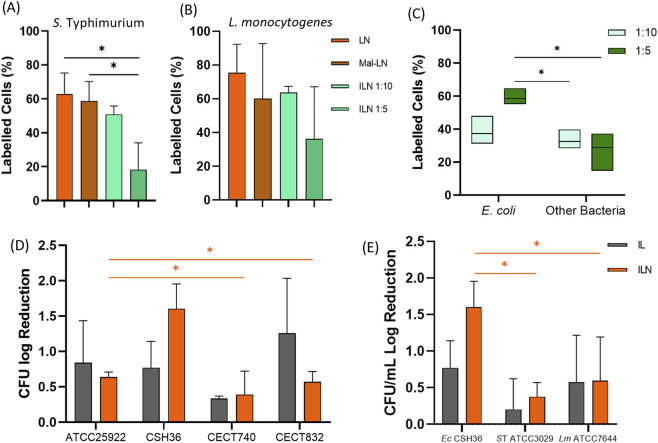
Comparison between the different LipoNAMs and ImmunoLipoNAMs formulations in terms of delivery efficiency of NAMs into **(A)** *Salmonella enterica* Typhimurium SGSC2523 and **(B)** *Listeria monocytogenes* ATCC7644. Percentage of cells exhibiting positive ACP1 staining determined by flow cytometry after 1 h; **(C)** Comparison of the efficiency of anti-*E. coli* ImmunoLipoNAMs against *Escherichia coli* and other bacterial species; **(D)** Effect of ImmunoLiposomes/ImmunoLipoNAMs with anti-*Escherichia coli* antibody at 1:5 antibody-maleimide molar ratio in the culturability of different *Escherichia coli* strains. **(E)** Effect of ImmunoLiposomes/ImmunoLipoNAMs with anti-*Escherichia coli* antibody at 1:5 antibody-maleimide molar ratio in the culturability of different foodborne pathogens (*Escherichia coli* CSH36, *Salmonella enterica* Typhimurium SGSC3029 and *Listeria monocytogenes* ATCC7644). *P < 0.05.

To determine if the incorporation of anti-*E. coli* antibodies influenced the specificity of the delivery of NAMs, the same assay was performed for two other foodborne pathogens: *Salmonella enterica* serovar Typhimurium SGSC2523 and *Listeria monocytogenes* ATCC7644 (Figures 7A–C). The inclusion of antibodies that do not target these species resulted in a decrease in the delivery of NAMs, as it decreases the electrostatic interactions between the cationic lipid DOTAP and the negatively charged LPS, while not adding affinity to an antigen on the cell surface. As expected, this decrease is dependent on antibody concentration, with the 1:5 antibody-maleimide ratio resulting in a more pronounced decrease in efficiency.

Finally, the effect of ImmunoLipoNAMs on the viability of several *E. coli strains* was evaluated for the best antibody-maleimide molar ratio (1:5), as determined by the flow cytometry assays. The influence of encapsulated NAMs is also evaluated by comparison with empty ImmunoLiposomes (Figure 7D). The absence of a consistently higher logarithmic reduction in CFU counts for ImmunoLipoNAMs suggests that cumulative lipid-mediated toxicity may be a primary driver of the observed decrease in cell culturability, potentially masking the specific antisense effect of the encapsulated NAMs.

The specificity of the *E. coli* targeted ImmunoLiposomes was also tested by assessing their antimicrobial activity (Figure 7E). The log reduction of CFU/mL was more significant in the *E. coli* than in the other tested foodborne pathogens, suggesting that the constructs have some degree of specificity towards the targeted bacteria.

### Citotoxicity of LipoNAMs/ImmunoLipoNAMs

To assess the *in vivo* applicability of the different formulations, their cytotoxicity was evaluated using the MTS cell viability assay in the HepG2 cell line and also and also *in vivo*, using the *Galleria mellonella* model, a widely used invertebrate *in vivo* model (Tsai et al., 2016).

Both antibody-maleimide molar ratios were tested, as well as the most efficient LipoNAM formulation (75:25 of DOTAP:DOPE with 1 mol% DSPE-methoxy-PEG) and an ImunoLipoNAM formulation tested beforehand by our research group (50:50 of DOTAP:DOPE with 3 mol% DSPE-methoxy-PEG and 2 mol% DSPE-maleimide-PEG and a 1:10 antibody-maleimide ratio) (Moreira et al., 2023). All the formulations were prepared with a 5 mM total lipid concentration and 500 nM of ACP1 ASO. Liposomal formulations without ASO were not tested, since the biocompatibility of this nucleic acid sequence had already been demonstrated (Moreira et al., 2023).

**FIGURE 8:**
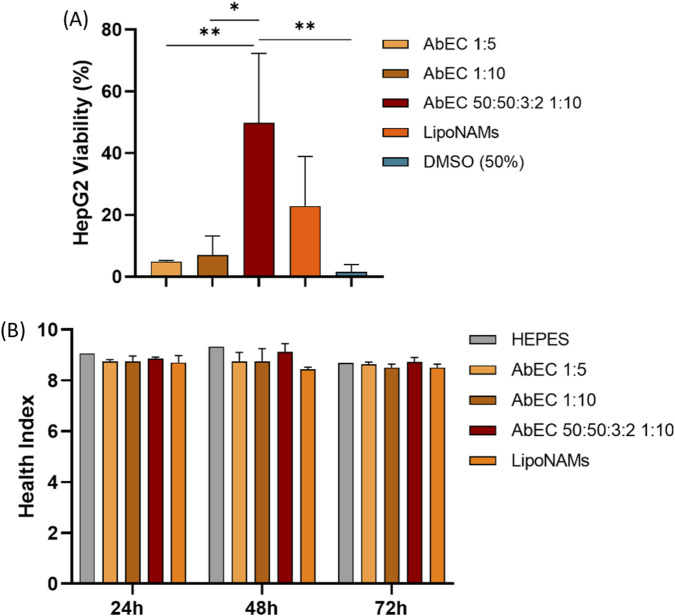
**(A)** Effect of ImmunoLipoNAMs with anti-*Escherichia coli* antibody and LipoNAMs (without antibodies) in the viability of the HepG2 cells. **(B)** Health index of *Galleria mellonela* after injection of the different formulations. *P < 0.05; **P < 0.01.

The previously tested ImmunoLipoNAMs formulation showed the best biocompatibility (near 50%), suggesting that the amount of cationic lipid is determinant for the interaction between liposomes and eukaryotic cells (Figure 8A). When testing formulations with higher DOTAP ratio, the best result (22.9% cell viability) is obtained for the LipoNAMs without antibody. Combining these results with the labeling of bacteria and the effect on culturability, it would appear that the application of antibodies did not yield the expected results, with the simpler formulation being more efficient across all the studied parameters.

These high cytotoxicity values can, however, be somewhat overestimated due to the direct contact between the HepG2 cells and the formulations (Allegra et al., 2018). This exposure, without any type of physiological barrier, does not occur during *in vivo* application.

Through the *G. mellonella* model (Tsai et al., 2016), the health index was assessed using a previously established health index score system (Loh et al., 2013), which quantifies different parameters such as survival, mobility, melanization and cocoon formation (Figure 8B). This method revealed no cytotoxicity for all tested formulations, with the health index maintaining the same value as the negative control, and a 100% survival probability 72 h after injection.

## Conclusion

The optimization of the liposomal delivery vector carried out in this work improved the previous formulation (with 50:50 DOTAP:DOPE ratio), with an increase of over 80% in labelling efficiency when 1 mol% of DSPE-methoxy-PEG was included. While the reductions in bacterial culturability hereby presented are still just on the limit of biological significance, this optimized formulation still represents progress in the field of ASObiotics.

Antibody conjugation to the liposomal surface imparted specificity to the formulation. Further characterization and optimization of ImmunoLipoNAMs is needed. Functionalization with different targeting molecules can also be tested in the near future, aiming for possible *in vivo* application without disrupting the natural microbiome and without cytotoxicity towards host cells.

## Data Availability

The original contributions presented in the study are included in the article/Supplementary Material, further inquiries can be directed to the corresponding author.
