# Supplementary material for: Liposomes as delivery vectors for nucleic acid mimics into Escherichia coli: effect of composition and functionalization
Source: Front Bioeng Biotechnol. 2026 May 28;14:1850483. doi: 10.3389/fbioe.2026.1850483 (PMC13253889; doi:10.3389/fbioe.2026.1850483)
Supplement: Supplementary file 1 [file DataSheet1.pdf]

Supplementary Information

Table S1. Loading efficiency (%) of LipoNAMs (500 nM ACP1) with different cationic and helper lipids at 75:25 molar ratio and 1 mol% DSPE-Methoxy-PEG.

	**Loaded ACP1 (Arbitrary Units)**	**Total ACP1 (Arbitrary Units)**	**Loading Efficiency (%)**
**DOTAP/DOPE**	43638	47146	92.6
	121047	148607	81.5
**DOTMA/DOPE**	48869	46898	≈100
	131245	134846	97.3
**DOTAP/CHEMS**	49644	45713	≈100
	138169	148854	92.8
**DOTAP/DPPE**	124669	128484	97.0
	134817	136335	98.9
**DOTMA/DPPE**	116039	127943	90.7
	129145	151606	85.2
**DOTMA/CHEMS**	120750	136527	88.4
	137532	149092	92.2
**DODAP/DOPE**	90318	91204	99.0
	88430	99141	89.2
**DODAP/CHEMS**	75989	81022	93.8
	86312	90202	95.7
**DODAP/DPPE**	73533	77361	95.1
	72882	82209	88.7

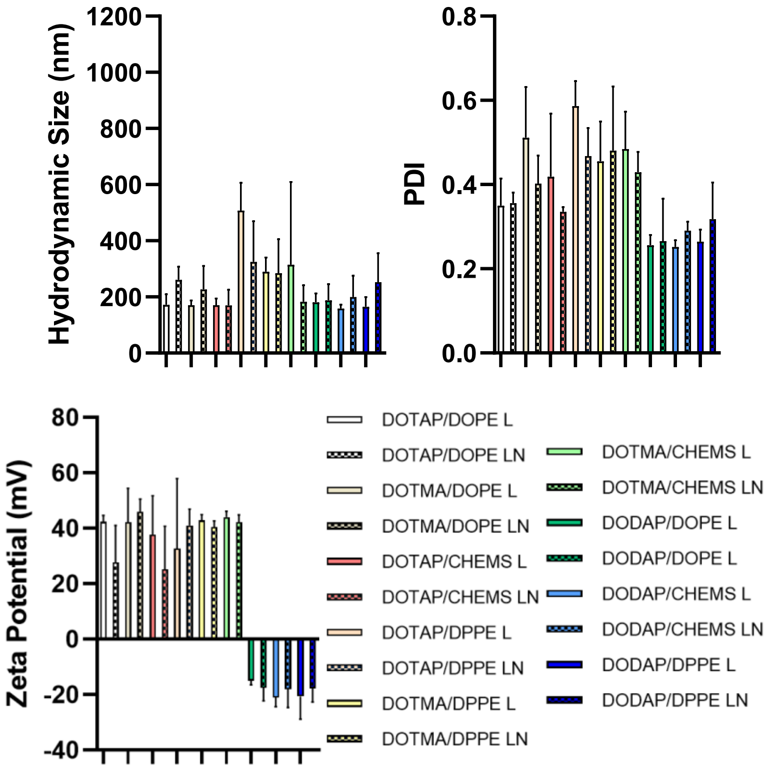

Figure S1. Physical properties of LipoNAMs (500 nM ACP1) with different cationic and helper lipids at 75:25 molar ratio and 1 mol% DSPE-methoxy-PEG.

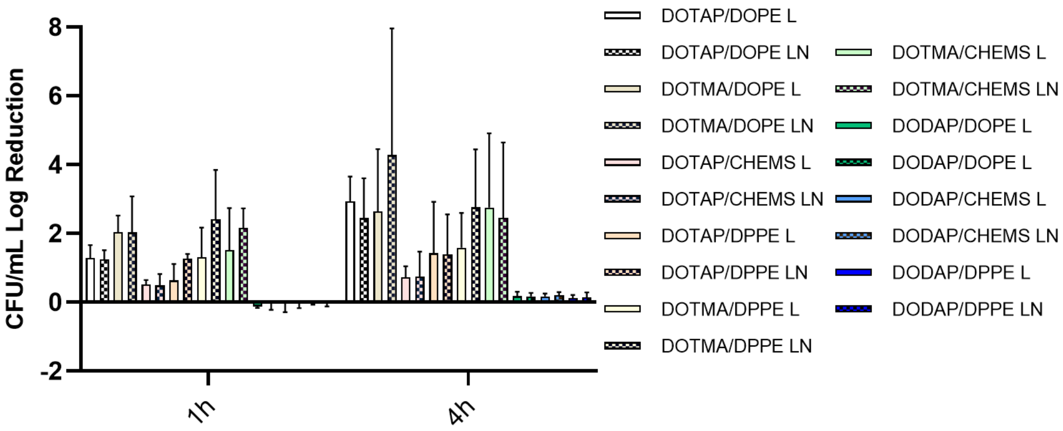

Figure S2. Culturability (expressed as CFU/mL log reduction) of *E. coli* ATCC25922 after 1h or 4h of exposure to different liposome and LipoNAM (500 nM ACP1) formulations (cationic/ionizable and helper lipids at 75:25 molar ratio and 1 mol% DSPE-methoxy-PEG).

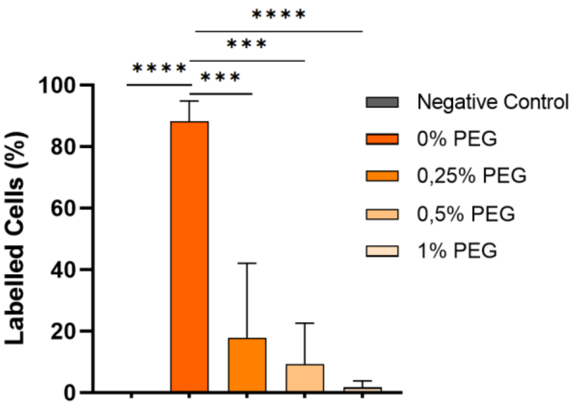

Figure S3. Percentage of *E. coli* cells exhibiting a positive ACP1 staining determined by flow cytometry after 1h in contact with LipoNAMs (500 nM ACP1) with 50:50 DOTAP:DOPE ratio and DSPE-methoxy-PEG in different percentages. *** P < 0.001; **** P < 0.0001.

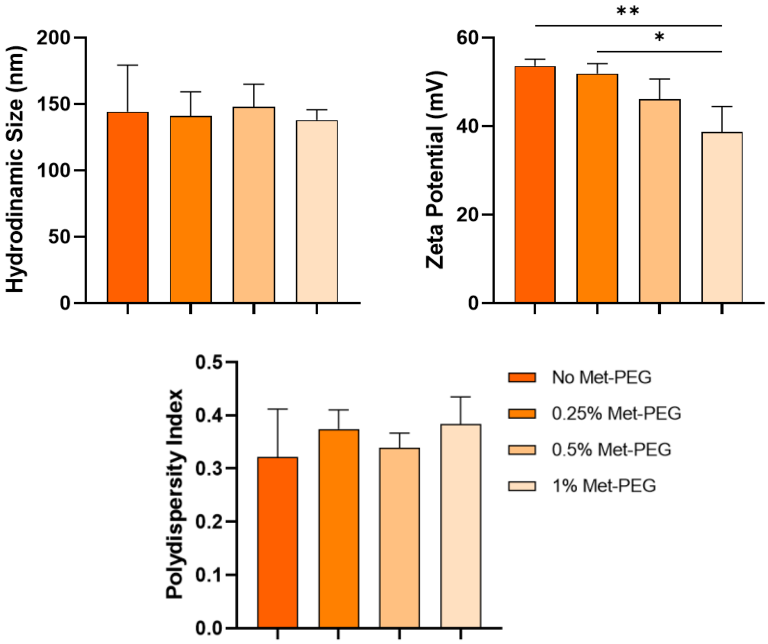

Figure S4. Physical properties of LipoNAMs with DOTAP and DOPE at 50:50 molar ratio and different molar percentages of DSPE-Methoxy-PEG. * P < 0.05; ** P < 0.01.

Table S2. Loading efficiency (%) of LipoNAMs with DOTAP and DOPE at 75:25 molar ratio and 1 mol% DSPE-Methoxy-PEG. The results were plotted as mean of 3 independent assays.

[ACP1] (nM)	Average Loading Efficiency (%)
250	99.7
500	97.7
1000	87.4

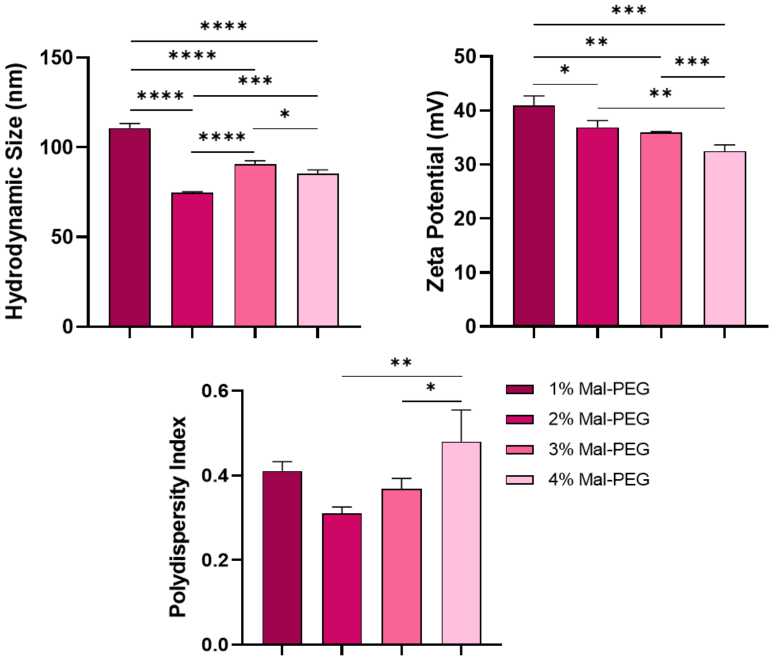

Figure S5. Physical properties of LipoNAMs with DOTAP and DOPE at 75:25 molar ratio, 1 mol% DSPE-Methoxy-PEG and different molar percentages of DSPE-Maleimide-PEG. * $P < 0.05$; ** $P < 0.01$; *** $P < 0.001$; **** $P < 0.0001$

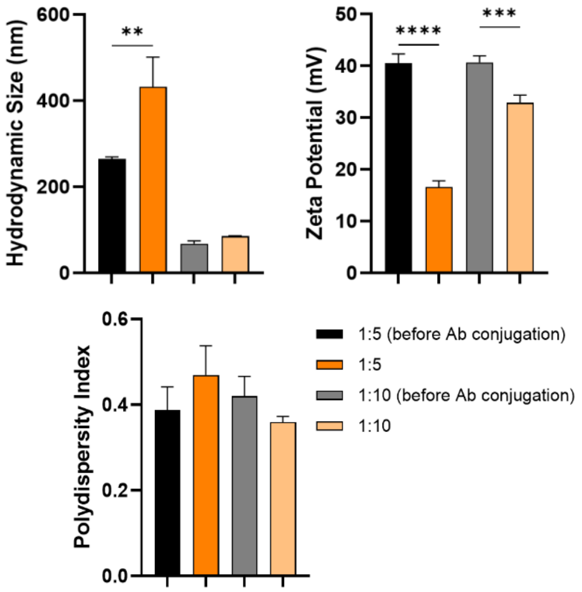

Figure S6. Physical properties of ImmunoLipoNAMs (AbEC) with DOTAP and DOPE at 75:25 molar ratio, 1 mol% DSPE-Methoxy-PEG, 1 mol% of DSPE-Maleimide-PEG and different antibody-maleimide ratios (1:5 or 1:10). The grey and black bars represent the LipoNAMs before antibody conjugation, for comparison. ** $P < 0.01$; *** $P < 0.001$; **** $P < 0.0001$

Table S3. *G. mellonella* health index scoring system, which assesses larval health based on survival, movement, cocoon formation and melanization, providing a semi-quantitative measure of infection severity and treatment efficacy[41].

Category	Status	Score
Survival	Dead	0
	Alive	2
Mobility	No movement	0
	Minimal movement upon stimulation	1
	Movement upon stimulation	2
	Movement without stimulation	3
Melanization	Black larvae	0
	Black spots on brown larvae	1
	≥ 3 spots on beige larvae	2
	< 3 spots on beige larvae	3
	No melanization	4
Cocoon Formation	No cocoon	0
	Partial cocoon	0,5
	Full cocoon	1
